# Analysis of the H-Ras mobility pattern *in vivo* shows cellular heterogeneity inside epidermal tissue

**DOI:** 10.1242/dmm.049099

**Published:** 2022-02-24

**Authors:** Radoslaw J. Gora, Babette de Jong, Patrick van Hage, Mary Ann Rhiemus, Fjodor van Steenis, John van Noort, Thomas Schmidt, Marcel J. M. Schaaf

**Affiliations:** 1 Animal Sciences and Health Cluster, Institute of Biology, Leiden University, Einsteinweg 55, 2333 CC Leiden, The Netherlands; 2 Biological, Soft and Complex Systems, Leiden Institute of Physics, Leiden University, Bohrweg 2, 2333 CA Leiden, The Netherlands

**Keywords:** H-Ras, Zebrafish, Single-molecule microscopy, Total internal reflection fluorescence microscopy, Membrane microdomains, Diffusion

## Abstract

Developments in single-molecule microscopy (SMM) have enabled imaging individual proteins in biological systems, focusing on the analysis of protein mobility patterns inside cultured cells. In the present study, SMM was applied *in vivo*, using the zebrafish embryo model. We studied dynamics of the membrane protein H-Ras, its membrane-anchoring domain, C10H-Ras, and mutants, using total internal reflection fluorescence microscopy. Our results consistently confirm the presence of fast- and slow-diffusing subpopulations of molecules, which confine to microdomains within the plasma membrane. The active mutant H-Ras^V12^ exhibits higher diffusion rates and is confined to larger domains than the wild-type H-Ras and its inactive mutant H-Ras^N17^. Subsequently, we demonstrate that the structure and composition of the plasma membrane have an imperative role in modulating H-Ras mobility patterns. Ultimately, we establish that differences between cells within the same embryo largely contribute to the overall data variability. Our findings agree with a model in which the cell architecture and the protein activation state determine protein mobility, underlining the importance of SMM imaging for studying factors influencing protein dynamics in an intact living organism.

This article has an associated First Person interview with the first author of the paper.

## Abstract (precis)

**Summary:** Single-molecule microscopy analysis of factors altering the *in vivo* dynamics of H-Ras proteins in epidermal cells in living zebrafish embryos revealed that cell architecture and protein activation state determine protein mobility.

## INTRODUCTION

Plasma membranes mostly consist of proteins and lipids that move laterally within the fluidic membrane plane and interact with each other in both random and organized manners (Singer and Nicolson, 1972). Until now, it has remained unclear what the origins are of many of such interactions and how the organization and mobility of all membrane constituents are governed (Gheber, 2018). Furthermore, not much is known about the role of structural complexes, such as the subjacent actomyosin cortex, in the dynamics of proteins and lipids in the plasma membrane. Subsequently, definitions of membrane domains and their sizes remain inconsistent. These domains include clathrin-coated pits and lipid rafts, and are believed to compartmentalize the membrane, facilitate the assembly of signaling complexes, and serve as the platforms for response amplification to the extracellular signaling molecules (Kusumi et al., 2012). For instance, it is now estimated that lipid rafts, cholesterol-enriched membrane domains, are predominantly transient complexes of 10-200 nm in size. Such lipid rafts are able to adjust their size and stability in response to ongoing membrane trafficking or signal transduction processes (Eggeling et al., 2009; Jacobson et al., 2019).

An important signaling protein that is present in the plasma membrane of many cell types in vertebrate organisms is H-Ras. The H-Ras protein is a member of the Ras protein family, which consists of small GTPases that activate intracellular signaling cascades, and thereby regulate crucial biological processes taking place in various cells, such as growth, proliferation and differentiation (Malumbres and Barbacid, 2003). Gain-of-function mutations in genes encoding Ras proteins are found in ∼25% of human cancers, which makes Ras proteins interesting targets of cancer therapies (Hobbs et al., 2016). These proteins are mainly localized at the plasma membrane, although some fractions have also been reported to be present in membranes of endosomes, the endoplasmic reticulum and Golgi apparatus. Various Ras isoforms exist, which differ predominantly in their so-called hyper-variable region (HVR), formed by 25 amino acids present in their carboxyl-terminal end. The most carboxyl-terminal part of the HVR comprises an anchoring domain, which is responsible for anchoring Ras proteins in the cytoplasmic leaflet of the cell membranes upon post-translational modifications, mostly the addition of lipid groups. In H-Ras, this domain comprises a CAAX motif, which can be farnesylated, and two cysteine residues that can be (reversibly) palmitoylated (Brunsveld et al., 2009; Willumsen et al., 1984).

Single-molecule microscopy (SMM) has been used in many studies to visualize individual molecules in the plasma membrane to study and characterize their organization and mobility. This technique is mostly performed using advanced fluorescence microscope techniques, such as light-sheet fluorescence microscopy or total internal reflection fluorescence microscopy (TIRFM). The used microscopy setups are equipped with a laser for excitation of the fluorescent molecules and with a highly sensitive charged-couple device (CCD) or a complementary metal oxide semiconductor camera for capturing the emitted photons. Molecules subjected to SMM experimentation are often fluorescently labeled. There are several options for selecting a suitable fluorescent label, based on the biological model used or a spectrum of excitation laser light available in the setup (Chudakov et al., 2010; Harms et al., 2001; Seefeldt et al., 2008). Most often, autofluorescent proteins, such as green (GFP) or yellow (YFP) fluorescent proteins fused with an endogenous protein, are used, as they are not toxic to living organisms and their use does not require permeabilization or fixation of the cells.

The mobility of Ras proteins or Ras membrane anchors fused to autofluorescent proteins has been studied and characterized by SMM in cultured cells with a positional accuracy of up to 30 nm and a temporal resolution in the span 5-50 ms (Harms et al., 1999; Lommerse et al., 2004, 2005). It was demonstrated that populations of H-Ras molecules in the plasma membrane segregate into a slow- and a fast-diffusing fraction. The slow-diffusing fraction of H-Ras proteins, which is also referred to as an immobile fraction because its displacements are very close to the positional accuracy, contains 10-40% of molecules depending on the cell type, and increases in size upon activation of H-Ras by administration of insulin or EGF to the cells, or by making a mutation in the protein that induces constitutive activation (e.g. as in H-Ras^V12^) (Lommerse et al., 2004, 2005; Murakoshi et al., 2004). Studies in which the H-Ras anchoring domain was fused with YFP (referred to as YFP-C10H-Ras) showed that the anchor exhibits similar mobility patterns to the non-activated, full-length H-Ras protein and to other anchors of human Ras proteins (e.g. K-Ras) (Lommerse et al., 2006).

For the purposes of research, cultured cells have the advantage of easier control and manipulation compared to living organisms. Nevertheless, experiments involving cultured cell models do not take into consideration the influence of cell-to-cell interactions and extracellular stimuli that are present within a tissue. Furthermore, they do not take into account factors an entire organism might be presented with that alter the context of the cells under investigation, such as changes due to the diurnal cycle or the response to a stressor. Therefore, in order to perform SMM studies with more translational value, we extended the applicability of the single-molecule research to a more physiologically relevant system, using the zebrafish embryo as a model organism for studying protein mobility patterns *in vivo*. Owing to their optical clarity, zebrafish embryos serve as excellent model organisms for visual analyses of physiological processes and for research using fluorescently labeled cells and proteins (Canedo and Rocha, 2021; Detrich et al., 2011; Garcia et al., 2016; Gore et al., 2018; Lieschke and Currie, 2007). The high fecundity and short generation time of the zebrafish facilitate genetic screens and identification of mutant phenotypes (Haffter et al., 1996; Reisser et al., 2018). In addition, genetically modified zebrafish embryos can be readily created by the use of microinjection techniques.

In several zebrafish models for cancer, oncogenic transformation of specific cell types is induced in embryos by overexpression of human H-Ras, in particular its constitutively active mutant H-Ras^V12^. For example, transformation of melanocytes and melanoma formation is induced upon H-Ras^V12^ overexpression in these cells (Feng et al., 2010; Michailidou et al., 2009; Santoriello et al., 2010), whereas H-Ras^V12^ overexpression in neural progenitor cells results in the formation of brain tumors (Mayrhofer et al., 2017). A similar, constitutively active mutant form of human K-Ras in myoblasts leads to the initiation of rhabdomyosarcoma (Langenau et al., 2007). Thus, the zebrafish embryo is a highly suitable model for studying the signaling pathways induced by human Ras proteins.

In a previous study, we used a TIRFM-based approach to perform SMM in zebrafish, and we analyzed the dynamics of YFP-C10H-Ras in epidermal cells of 2-day-old embryos. The observed mobility patterns in the zebrafish embryos were different from those found in cultured cells, which underlined the importance of performing this type of study *in vivo*. Therefore, in the present study, we extended this application of the *in vivo* SMM technique to the full-length H-Ras protein. In addition to the wild-type H-Ras, we used a constitutively active and inactive H-Ras mutant (H-Ras^V12^ and H-Ras^N17^, respectively) to examine how the protein activity influences the patterns of diffusion and confinement of the H-Ras molecules. Furthermore, we studied the alterations in the mobility pattern during embryonic development and after treatment with Latrunculin B (LatB), a chemical inhibitor of actin polymerization, and with methyl-β-cyclodextrin (MBCD), which disrupts membrane organization by depleting cells of cholesterol. Ultimately, we performed experiments with YFP-C10H-Ras and the full-length H-Ras in human embryonic kidney cells (HEK293T) to compare the results obtained in the zebrafish embryos with results obtained in cultured cells using the same experimental protocol.

Our findings reveal that, for YFP-C10H-Ras, YFP-H-Ras, YFP-H-Ras^V12^ and YFP-H-Ras^N17^, in epidermal cells of the zebrafish embryos and in cultured HEK293T cells, a slow- and a fast-diffusing population of molecules can be distinguished and that both populations show confined diffusion. Differences in the fast-diffusing fraction initial diffusion coefficients and confinement sizes are also detected between the full-length, wild-type H-Ras and its constitutively active, oncogenic mutant, H-Ras^V12^. In addition, we show that treatment with both LatB and MBCD significantly influences values of initial diffusion coefficients as well as sizes of confinement area, pointing to a dominant role of plasma membrane composition in regulating H-Ras protein dynamics. Interestingly, in zebrafish embryos, the mobility pattern does not change during embryonic development and the variability between individual embryos is smaller than the variability between different experimental days and between different areas in the epidermis of one embryo.

## RESULTS

### Mobility patterns of YFP-C10H-Ras and YFP-H-Ras in HEK293T cells

As an initial step in our SMM study, the YFP fusion proteins of the H-Ras anchoring domain and full-length H-Ras protein (YFP-C10H-Ras and YFP-H-Ras, respectively) were studied in cultured HEK293T cells using our TIRFM setup. These experiments aimed to compare findings obtained using the TIRFM setup with data previously obtained in cell cultures (Bobroff, 1986; Lommerse et al., 2005), and with our previous findings in zebrafish embryos (Schaaf et al., 2009). Prior to the SMM imaging, cells were transiently transfected with YFP-C10H-Ras and YFP-H-Ras expression vectors and screened in order to analyze the expression levels and subcellular localization of the fluorescent proteins. Images of HEK293T cells expressing YFP-C10H-Ras, obtained through confocal laser scanning microscopy, indicated predominant membrane localization of the signal coming from the YFP fused with the C10H-Ras membrane anchor, which is in line with patterns observed before in mouse fibroblast and human embryonic kidney cells (3T3-A14 and ts201, respectively) (Lommerse et al., 2004, 2005).

**Fig. 1.**
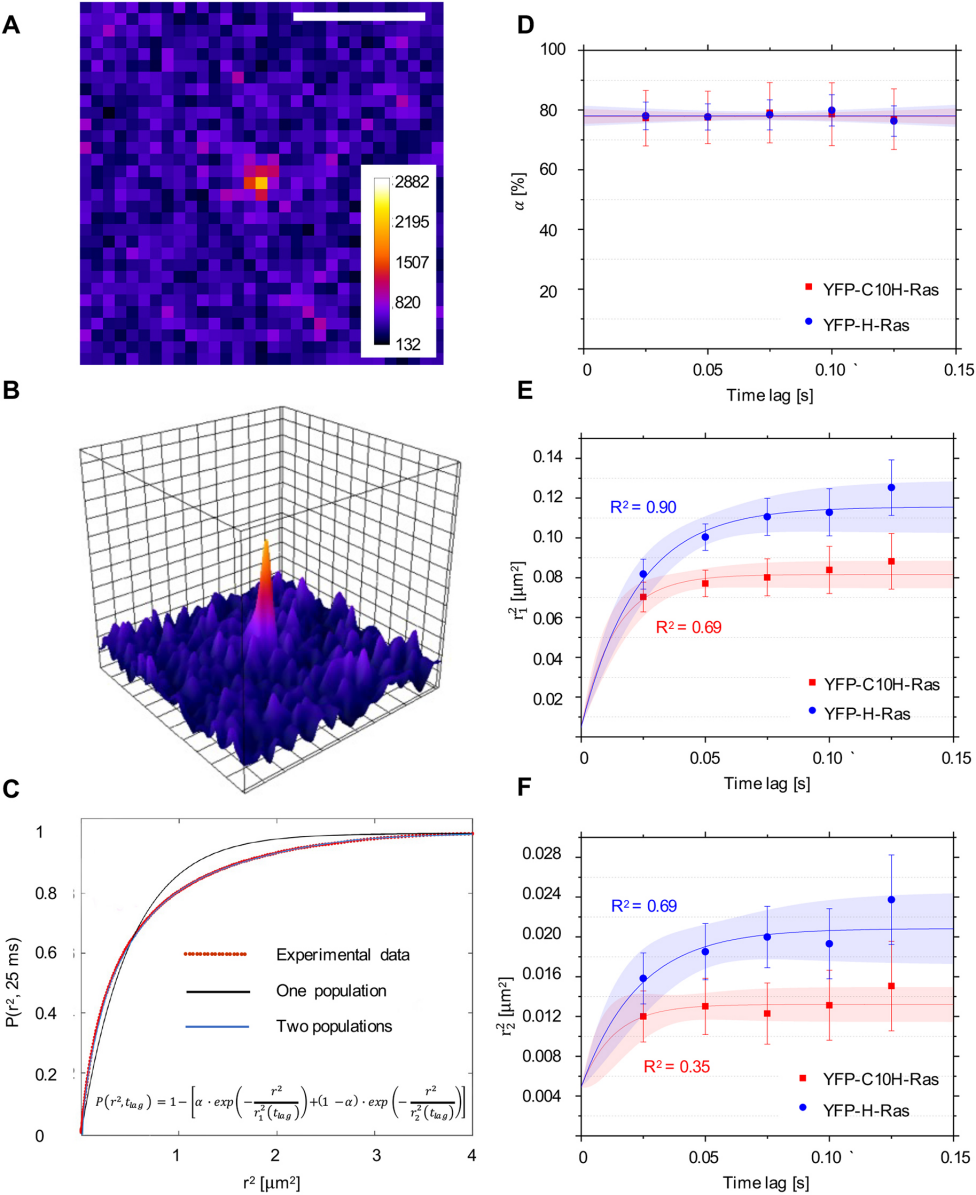
**Single-molecule microscopy (SMM) analysis: protein localization and mobility patterns of YFP-C10H-Ras and YFP-H-Ras in HEK293T cells.** (A) SMM intensity map showing signal of a YFP-C10H-Ras molecule located in the basal membrane of a HEK293T cell. Scale bar: 2 µm. (B) Three-dimensional representation of the image shown in A, depicting fluorescence intensities of each pixel. One visible intensity peak is shown that is attributed to a single YFP-C10H-Ras molecule. Over these peaks, two-dimensional Gaussian surfaces were fitted. (C) Representative cumulative distribution plot of squared displacements determined using particle image correlation spectroscopy (PICS) analysis. Data points are shown in red, and the two populations biexponential model that best fits the data points in blue (formula shown). Fitting of the data points to the two populations model allows for calculation of a relative size of the subpopulations (α) and their mean squared displacements (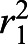 and 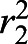). This procedure was repeated for each of the time lags used. (D) Fraction size of the fast-diffusing population (α), plotted against the time lag. (E) Mean squared displacements plotted against the time lag for the fast-diffusing fraction 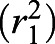. (F) Mean squared displacements plotted against the time lag for the slow-diffusing fraction 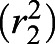. Results of the fits are summarized in Table 1. To establish the values of dynamic parameters, at least three individual HEK293T cells per H-Ras construct (YFP-C10H-Ras and YFP-H-Ras) were imaged on each of the three different experimental days. Each data point is presented in the form of a mean±s.e.m., and the 95% confidence interval (c.i.) of the mathematical fit is shown. Shapiro–Wilk statistical test was performed to check for normality of the data set. Statistical analysis was performed using an unpaired Student's *t*-test with the resulting *P*-value *P*(α, 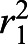, 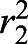)>0.05 at a *t*_lag_ of 25 ms. Pearson correlation coefficients (*R*^2^) are presented to show fitness of the data to the model of confined diffusion.

Three days after transfection, YFP-C10H-Ras and YFP-H-Ras expression levels in HEK293T cells were significantly decreased. The sparse distribution allowed for identification of single YFP-C10H-Ras molecules in the basal cell membrane, using our TIRFM setup. Diffraction-limited fluorescence intensity peaks were observed, and Gaussian curves were fitted over these peaks (Fig. 1A,B). Full width at half maximum (FWHM) and intensity values of the Gaussian distributions corresponding to signals from single YFP molecules were established in fixed HEK293T cells, taking into consideration only the molecules that displayed single-step photobleaching, indicating that their signal originated from an individual YFP molecule. These values were subsequently used to determine cutoff values for peak selection. The signal-to-noise ratio, defined as the quotient between the average intensity of a single fluorophore (2215 counts) and the standard deviation of the background signal (74 counts), approximated to 30. The positional accuracy equaled approximately 22 nm in one dimension for the localization of these single molecules. Image sequences were acquired using a time lag of 25 ms, and the mobility pattern of the proteins was determined by particle image correlation spectroscopy (PICS) software (Semrau and Schmidt, 2007). Using a multistep analysis, information was yielded for five different time lags: 25, 50, 75, 100 and 125 ms. By means of the PICS software, correlations between the locations of molecules in consecutive frames were determined, and cumulative probability distributions of the displacements were generated for each time lag. The data were then fitted to a one- or two-population model (Fig. 1C). The two-population model fitted significantly better, indicating the presence of two fractions of molecules with different mobility patterns. Using these curves, the relative size of the fast-diffusing fraction (α) and the mean squared displacements of the fast and slow diffusing fractions (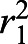 and 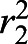, respectively) were established for each time lag.

**Table 1.**
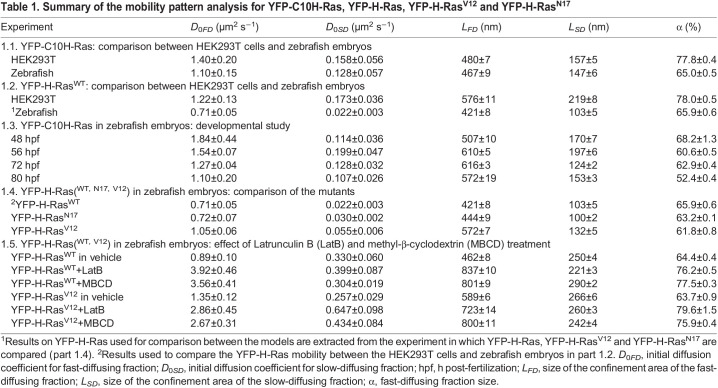
Summary of the mobility pattern analysis for YFP-C10H-Ras, YFP-H-Ras, YFP-H-Ras^V12^ and YFP-H-Ras^N17^

Subsequently, for both YFP-C10H-Ras and YFP-H-Ras, the parameters α, 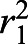 and 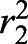 were plotted against the chosen time lags (Fig. 1D-F). The relative size of the fast-diffusing fraction α was constant over all time lags (Fig. 1D), and equaled 77.8±0.4% and 78.0±0.5% for YFP-C10H-Ras and YFP-H-Ras, respectively. The curves presenting the relationship between mean squared displacements (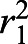 and 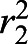) and the time lag for the fast- and slow-diffusing fractions are non-linear and reach a plateau (Fig. 1E,F). We therefore fitted these curves using a confined diffusion model, in which the movement of YFP-C10H-Ras and YFP-H-Ras is confined within an area of size *L* with an initial diffusion coefficient *D*_0_ (Bobroff, 1986; Lingwood and Simons, 2010; Schaaf et al., 2009). The mobility patterns of the two fusion proteins were remarkably similar. For YFP-C10H-Ras, *D*_0_ of the fast-diffusing fraction equaled 1.40±0.20 µm^2^ s^−1^, and the size of the confinement area *L* equaled 480±7 nm. For YFP-H-Ras, the fast-diffusing fraction had a *D*_0_ of 1.22±0.13 µm^2^ s^−1^ and was confined in an area in which *L* equaled 576±11 nm. The slow-diffusing fractions of YFP-C10H-Ras and YFP-H-Ras moved dramatically slower (*D*_0_ of 0.158±0.056 µm^2^ s^−1^ and 0.173±0.036 µm^2^ s^−1^, respectively) and were confined in smaller areas (*L* of 157±5 nm and 219±8 nm, respectively).

### Mobility patterns of YFP-C10H-Ras and YFP-H-Ras in epidermal cells of zebrafish embryos

**Fig. 2.**
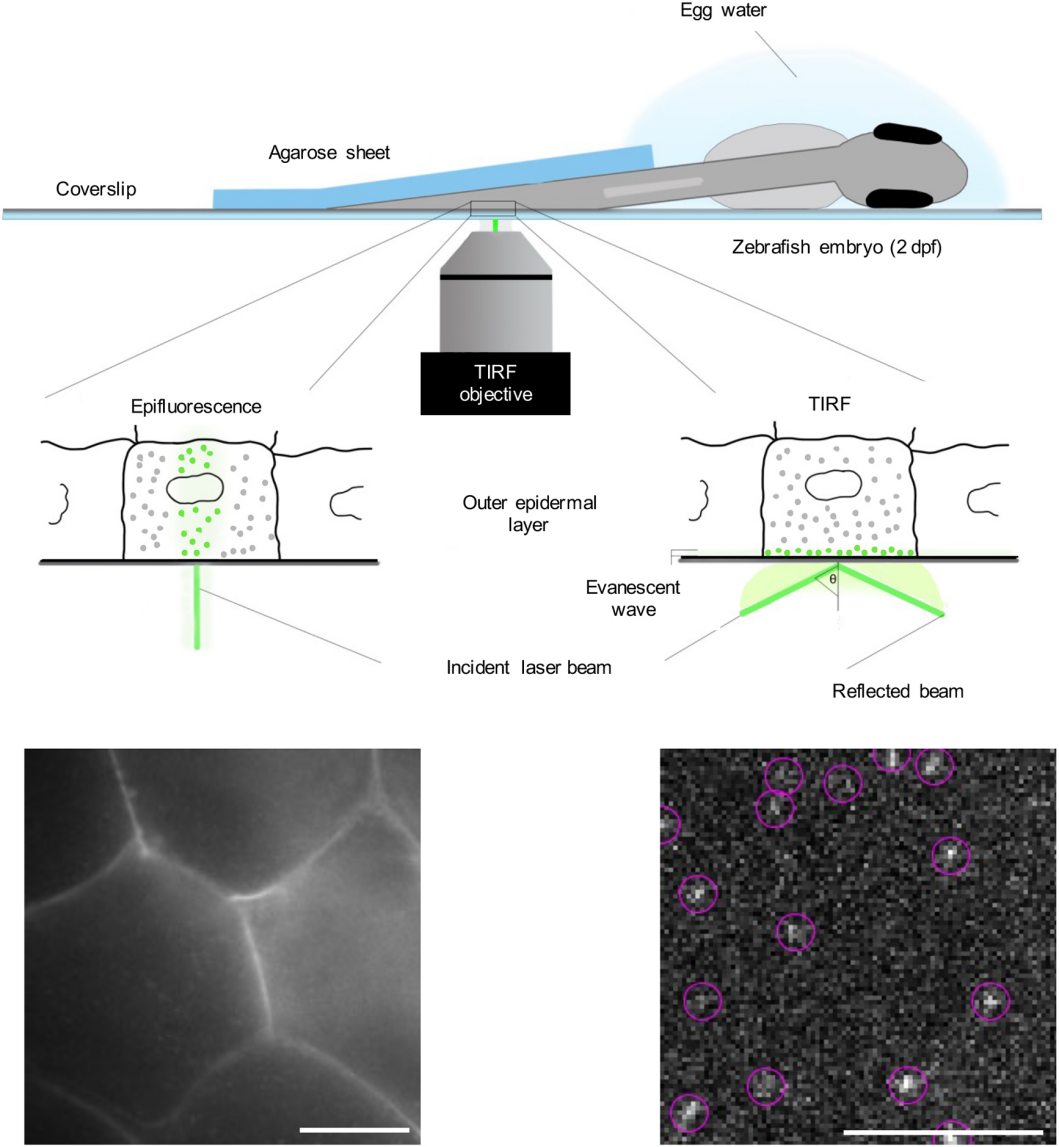
**Schematic overview of SMM applied to a living zebrafish embryo.** A zebrafish embryo was injected with an expression vector for a YFP fusion protein shortly after fertilization. At 2 dpf, it was placed on a coverslip coated with poly-L-lysine in a drop of egg water. The tail region of the embryo was covered with a 0.75-mm-thick agarose (2%) sheet. On the lower left part of the figure, an epifluorescence picture is presented of the outer layer of the epidermis, showing the fluorescent signal of YFP-C10H-Ras in the cell membranes. Morphologically, the cells in this layer are homogenous and are characterized by a hexagonal shape. On the lower right part, a TIRFM image of an area within the field of view of the epifluorescence picture is presented, with examples of individual YFP molecules shown in circles. Scale bars: 5 µm.

**Fig. 3.**
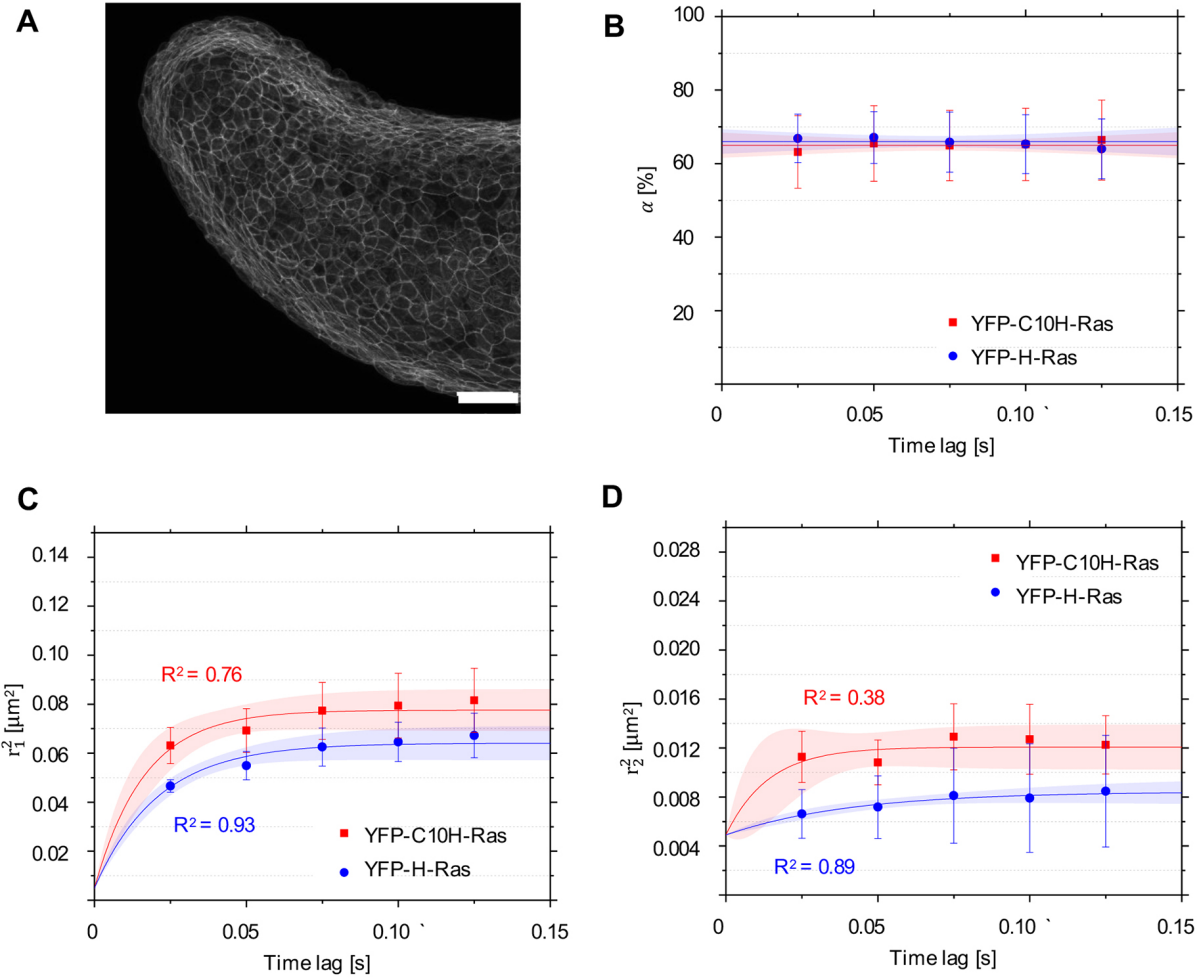
**Mobility patterns of YFP-C10H-Ras and YFP-H-Ras in epidermal cells of the zebrafish embryos.** (A) Confocal microscopy image of the outer epidermal cell layer of a 2 dpf zebrafish embryo from the transgenic line *Tg(bactin:GFP-C10H-Ras)^vu119^*, showing the fluorescence signal from GFP-C10H-Ras. Scale bar: 50 µm. (B) Fraction size of the fast-diffusing population (α), plotted against the time lag. (C) Mean squared displacements plotted against the time lag for the fast-diffusing fraction 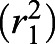. (D) Mean squared displacements plotted against the time lag for the slow-diffusing fraction 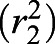. Results of the fits are summarized in Table 1. To establish the values of dynamic parameters, three different embryos per each H-Ras construct (YFP-C10H-Ras and YFP-H-Ras) were imaged on each of the three (six for YFP-C10H-Ras) different experimental days. Each data point is presented in the form of mean±s.e.m., and the 95% c.i. of the mathematical fit is shown. Shapiro–Wilk statistical test was performed to check for normality of the data set. Statistical analysis was performed using an unpaired Student's *t*-test with a resulting *P*-value of *P*(α, 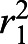, 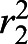)>0.05 at a *t*_lag_ of 25 ms. Pearson correlation coefficients (*R*^2^) are presented to show fitness of the data to the model of confined diffusion.

In order to successfully image membrane proteins in living zebrafish embryos, we focused our observation on the outer epidermal cell layer in the tail fin of 2 days-post-fertilization (dpf) zebrafish embryos (Fig. 2). Embryos were injected at the one-cell stage with DNA constructs encoding YFP-C10H-Ras and YFP-H-Ras in order to transiently express the YFP-tagged H-Ras anchor and the full-length H-Ras protein in the zebrafish tail fin. This outer cell layer of the skin (the superficial stratum) is a homogenous layer of hexagonal cells (Fig. 2, epifluorescence mode) and forms the upper part of the skin (the epidermis) together with an underlying cell layer. The localization of GFP-C10H-Ras in the cells in this layer is illustrated by a representative confocal microscopic image of a 1 dpf embryo taken from a transgenic zebrafish line expressing this fusion protein in all cells (Fig. 3A).

The tail fins of 2 dpf zebrafish embryos are morphologically stable enough to resist coverage with a 0.75-mm-thick sheet of agarose, which was used in order to gently press the tail fin towards the surface. This enables the evanescent field to excite the fluorophores present in the outer membrane of cells in the outer epidermal cell layer. For imaging, we focused on the tail fin region of the zebrafish embryo, while the rest of the zebrafish body was immersed in a drop of water. Zebrafish vital functions, such as heartbeat and the blood flow in the cardiovascular system, were checked under a stereofluorescence microscope post-imaging.

As in HEK293T cells, we observed fast- and slow-diffusing protein fractions in zebrafish embryos (Fig. 3B). The fast-diffusing fraction size α equaled 65.0±0.5% for YFP-C10H-Ras and 65.9±0.6% for YFP-H-Ras. In both of the fractions, molecules followed a confined diffusion pattern (Fig. 3C,D), with sizes of the confinement areas *L* for the fast-diffusing fractions being approximately three to four times larger than those for the slow-diffusing ones (see Table 1, part 1.1 and 1.2). The initial diffusion coefficient *D*_0_ of the fast-diffusing fraction equaled 1.10±0.15 µm^2^ s^−1^ for YFP-C10H-Ras and 0.71±0.05 µm^2^ s^−1^ for YFP-H-Ras. The initial diffusion coefficients for the slow-diffusing fractions were at least eight times lower than the ones for their fast-diffusing counterparts. When we compared these data to those obtained in the HEK293T cells, we found a significant difference between the mobility patterns of YFP-H-Ras in HEK293T cells and those in epidermal cells of zebrafish embryos. The initial diffusion coefficients *D*_0_ of both the fast- and the slow-diffusing fractions were significantly lower in the zebrafish embryonic cells (see Table 2). No significant differences were found between the YFP-C10H-Ras mobility patterns in HEK293T cells and in zebrafish embryos.

**Table 2.**
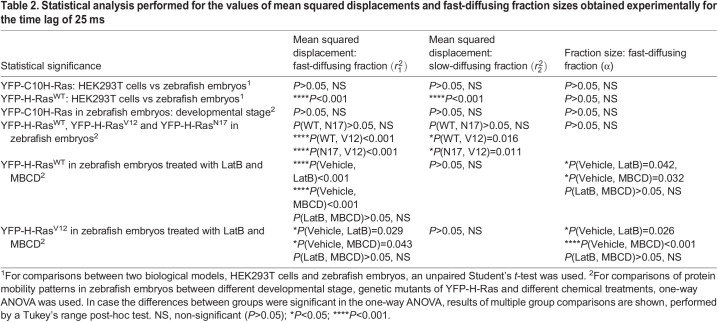
Statistical analysis performed for the values of mean squared displacements and fast-diffusing fraction sizes obtained experimentally for the time lag of 25 ms

### Mobility patterns of YFP-C10H-Ras in zebrafish embryos at different developmental stages

To study the stability of the data obtained in the zebrafish embryos over different developmental stages, we analyzed the mobility patterns of YFP-C10H-Ras at 48, 56, 72 and 80 h post-fertilization (hpf). At each stage, the values for the parameters α, 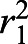 and 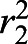 were determined for each time lag, and *D*_0_ and *L* were determined for both fast- and slow-diffusing populations (Table 1, part 1.3; Fig. S1). The results showed that differences in the developmental stage of the zebrafish embryo did not significantly influence any of these parameters (Table 2). This means that the C10H-Ras mobility patterns remain stable within the time frame of our experiments.

### Mobility patterns of YFP-H-Ras^V12^ and YFP-H-Ras^N17^ in epidermal cells of zebrafish embryos

**Fig. 4.**
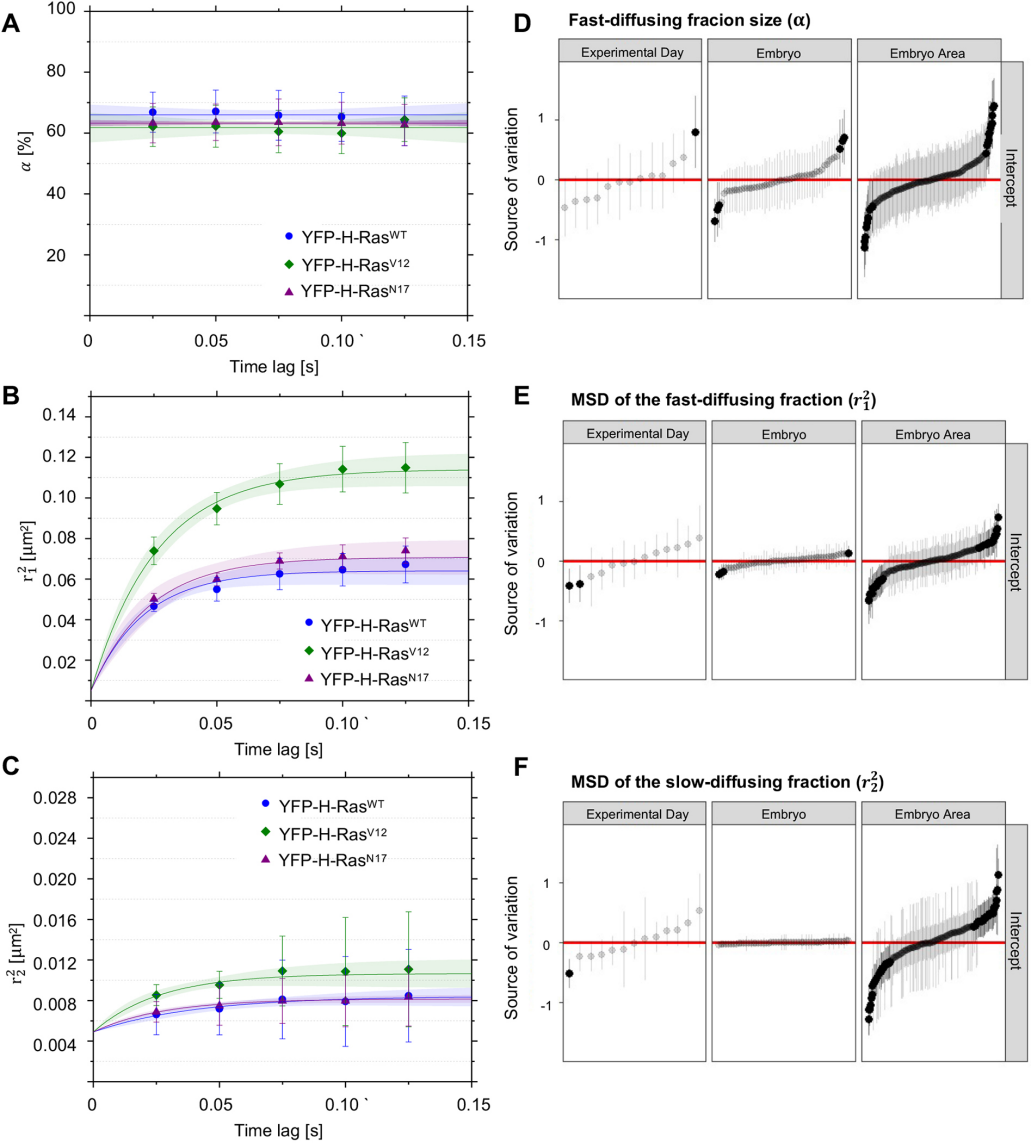
**Mobility patterns of YFP-H-Ras and its mutants, YFP-H-Ras^V12^ and YFP-H-Ras^N17^, in epidermal cells of the zebrafish embryos.** (A) Fraction size of the fast-diffusing population (α), plotted against the time lag. (B) Mean squared displacements plotted against the time lag for the fast-diffusing fraction 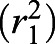. (C) Mean squared displacements plotted against the time lag for the slow-diffusing fraction 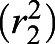. Results of the fits are summarized in Table 1. To establish the values of dynamic parameters, three different embryos per each H-Ras genetic mutant (YFP-H-Ras, YFP-H-Ras^V12^ and YFP-H-Ras^N17^) were imaged on each of the three different experimental days. Each data point is presented in the form of a mean±s.e.m., and the 95% c.i. of the mathematical fit is shown. Shapiro–Wilk statistical test was performed to check for normality of the data set. Statistical analysis was performed, using a one-way ANOVA [*P*(α)>0.05 and *P*(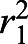, 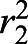)<0.05 at a *t*_lag_ of 25 ms] with a Tukey's range post-hoc test (for details of the post-hoc test results, see Table 2). (D-F) Caterpillar plots presenting the effect range of a different experimental day, different embryos within an experimental day, and different areas within an embryo to an overall variability in the fast-diffusing fraction size (D), mean squared displacement of the fast-diffusing fraction (E) and mean squared displacement of the slow-diffusing fraction (F). Effect ranges represent the relative deviation of group intercepts from the overall mean with 95% c.i. Red lines indicate the overall mean of the data, while black points denote groups that significantly deviate from the overall mean among experimental days, individual embryos and areas within an embryo. The data points are sorted based on their deviation from the total average, beginning with the ones most negatively deviating from the overall mean. The values in D were logit transformed, and the values in E and F were logarithm transformed, to meet the statistical hypotheses of the hierarchical linear model. MSD, mean squared displacement.

To study the effect of the activation state of the H-Ras protein, the constitutively active H-Ras^V12^ mutant (with a valine replacing a glycine at position 12) and the inactive mutant H-Ras^N17^ (with an asparagine replacing a serine at position 17) were used. The fusion proteins YFP-H-Ras, YFP-H-Ras^V12^ and YFP-H-Ras^N17^ were expressed in 2 dpf zebrafish embryos, and the mobility patterns of the each of these constructs were analyzed. Again, we observed fast- and slow-diffusing fractions of molecules. The size of the fast-diffusing fraction did not differ significantly between the constructs, and equaled 66.0±0.5% for YFP-H-Ras, 61.7±0.7% for YFP-H-Ras^V12^ and 63.2±0.2% for YFP-H-Ras^N17^ (Fig. 4A). All fractions showed confined diffusion. Initial diffusion coefficients *D*_0_ for the fast-diffusing fraction equaled 0.70±0.05 µm^2^ s^−1^ for YFP-H-Ras, 1.05±0.06 µm^2^ s^−1^ for YFP-H-Ras^V12^ and 0.72±0.07 µm^2^ s^−1^ for YFP-H-Ras^N17^, with the diffusion coefficient for YFP-H-Ras^V12^ being significantly higher than those for the other two constructs (Fig. 4B). Similarly, the size of the confinement area *L* for YFP-H-Ras^V12^ (572±7 nm) was significantly higher than those for YFP-H-Ras and YFP-H-Ras^N17^ (421±8 nm and 444±9 nm, respectively). The initial diffusion coefficients and the sizes of the confinement areas for the slow-diffusing fractions of YFP-H-Ras^V12^ and YFP-H-Ras^N17^ did not show any significant difference from the values determined for the wild-type YFP-H-Ras (0.022±0.003 µm^2^ s^−1^ and 103±5 nm; for detailed results, see Table 1, part 1.4, Table 2, Fig. 4C).

### Mobility patterns of YFP-H-Ras and YFP-H-Ras^V12^ in epidermal cells of zebrafish embryos after treatment with LatB and MBCD

**Fig. 5.**
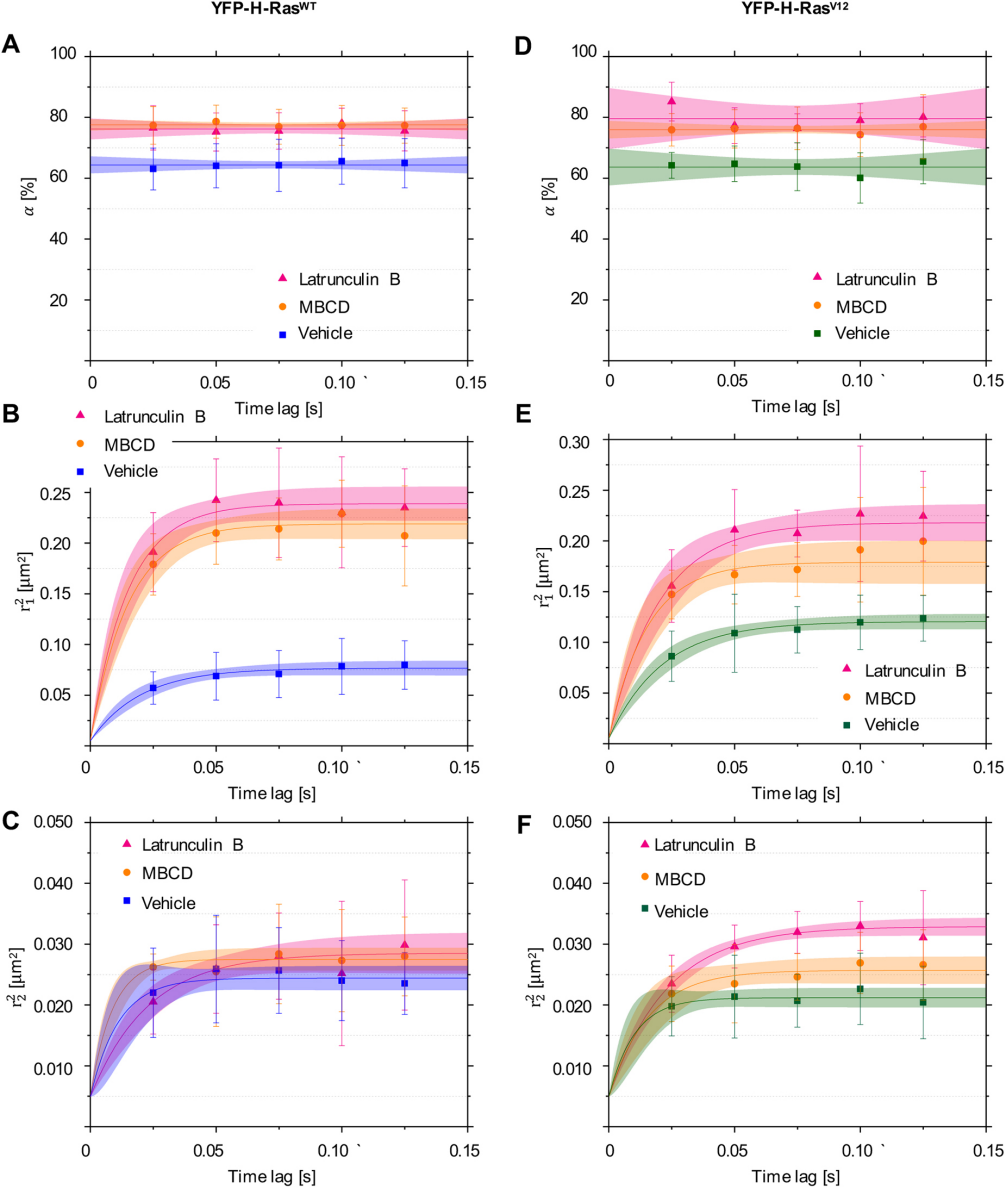
**Mobility patterns of YFP-H-Ras and the constitutively active mutant YFP-H-Ras^V12^, in epidermal cells of the zebrafish embryos after treatment with Latrunculin B (LatB) and methyl-β-cyclodextrin (MBCD).** (A-C) Plots representing mobility patterns of the wild-type YFP-H-Ras. (A) Fraction size of the fast-diffusing population (α), plotted against the time lag. (B) Mean squared displacements plotted against the time lag for the fast-diffusing fraction 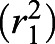. (C) Mean squared displacements plotted against the time lag for the slow-diffusing fraction 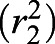. (D-F) Plots representing mobility patterns of the constitutively active YFP-H-Ras^V12^. (D) Fraction size of the fast-diffusing population (α), plotted against the time lag. (E) Mean squared displacements plotted against the time lag for the fast-diffusing fraction 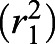. (F) Mean squared displacements plotted against the time lag for the slow-diffusing fraction 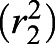. Parameters obtained upon curve fitting are presented in Table 1. To establish the values of dynamic parameters, three different embryos per each treatment group (vehicle, LatB and MBCD) and construct (YFP-H-Ras^V12^, YFP-H-Ras) were imaged on each of the three different experimental days. Each data point is presented in the form of a mean±s.e.m., and the 95% c.i. of the mathematical fit is shown. Shapiro–Wilk statistical test was performed to check for normality of the data set. Statistical analysis was performed using a one-way ANOVA [for both H-Ras^WT^ and H-Ras^V12^ *P*
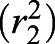
>0.05 and *P*(α, 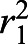)<0.05 at a *t*_lag_ of 25 ms] with a Tukey's range post-hoc test (for details of the post-hoc test results, see Table 2).

In order to understand how actin microfilaments and cholesterol affect H-Ras mobility, 2-day-old zebrafish larvae expressing YFP-H-Ras or its constitutively active mutant, YFP-H-Ras^V12^, were incubated with LatB or MBCD. After treatment with LatB or MBCD, SMM was performed, and we observed a fast- and a slow-diffusing fraction of molecules in all experimental groups. Interestingly, the size of the fast-diffusing fractions significantly increased after LatB and MBCD treatment for YFP-H-Ras (from 64.4±0.4% to 76.2±0.5% and 77.5±0.3% after LatB and MBCD treatment, respectively) (Fig. 5A) and YFP-H-Ras^V12^ (from 63.7±0.9% to 79.6±1.5% and 75.9±0.4% after LatB and MBCD treatment, respectively) (Fig. 5D). Additionally, treatment with LatB and MBCD significantly increased the initial diffusion coefficients and the sizes of the confinement areas of the fast-diffusing fraction for both YFP-H-Ras and YFP-H-Ras^V12^ (Fig. 5B,E). For YFP-H-Ras, treatment with LatB increased this diffusion coefficient from 0.89±0.10 µm^2^ s^−1^ to 3.92±0.46 µm^2^ s^−1^, while treatment with MBCD changed it to 3.56±0.41 µm^2^ s^−1^. The size of the confinement area of the fast fraction grew from 462±8 nm to 837±10 nm after LatB treatment and to 801±9 nm after MBCD treatment (Table 1, part 1.5, Fig. 5B). For the YFP-H-Ras^V12^ mutant, treatment with LatB increased the diffusion coefficient of the fast-diffusing fraction from 1.35±0.12 µm^2^ s^−1^ to 2.86±0.45 µm^2^ s^−1^, while treatment with MBCD altered it to 2.67±0.31 µm^2^ s^−1^. The size of the YFP-H-Ras^V12^ fast fraction's confinement area increased from 589±6 nm to 723±14 nm after LatB treatment and to 800±11 nm after MBCD treatment (Table 1, part 1.5, Fig. 5E). Such changes, however, were not observed in the slow-diffusing fractions, both for YFP-H-Ras and YFP-H-Ras^V12^ (Fig. 5C,F). Taken together, these data show that treatment with LatB and MBCD abolished differences between H-Ras and H-Ras^V12^ dynamics by increasing the diffusion coefficient and confinement area of the fast-diffusing fractions.

### Sources of variability in the results

Finally, we analyzed the sources of variability in the results obtained in zebrafish embryos for YFP-H-Ras and the two mutants, YFP-H-Ras^V12^ and YFP-H-Ras^N17^. For this purpose, we first studied the correlation between the number of individual molecules within images and the parameters α, 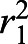 and 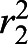. Correlation coefficients between the number of molecules and the corresponding parameters were low (0.22, 0.19 and 0.28, respectively), indicating that the correlation between the number of molecules in an image and the parameters describing protein mobility is very weak. Thus, we report that the number of molecules has a negligible impact on the H-Ras mobility analysis.

Second, the contribution of three factors to the overall variability in the results was analyzed. These factors were different experimental days, different individual embryos and different areas imaged within a single embryo. By use of hierarchical linear mixed models, we generated caterpillar plots for α, 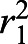 and 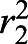, which present random effects distribution, being the deviation of the group intercept from an overall mean, and the contribution of each of the three factors to the overall mean of a given parameter (Fig. 4D-F). Subsequently, we quantified the percentage contribution of each of the selected sources of variation towards the overall data variability (Table 3). These caterpillar plots and the quantitative source of variation analysis both show that, for every parameter, most of the random effects come from imaging embryos on different experimental days (contribution to the total variability of 33.1% for the fraction size, 49.2% for the fast-diffusing fraction diffusion coefficient) and imaging different areas within the epidermis of the same zebrafish embryo (contribution of 50.1% for the slow-diffusing subpopulation diffusion coefficient). Interestingly, imaging different embryos from the same zebrafish batch was the smallest source of variability for all parameters and did not introduce nearly as much variability as imaging different areas within the same individual embryo (Table 3).

**Table 3.**
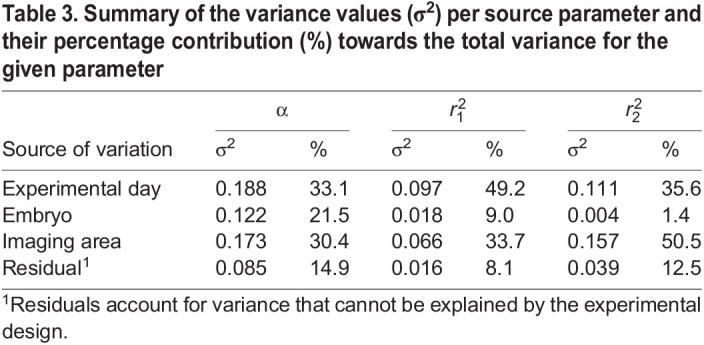
Summary of the variance values (σ^2^) per source parameter and their percentage contribution (%) towards the total variance for the given parameter

## DISCUSSION

In the present study, we have applied SMM to an *in vivo* model system, using living zebrafish embryos. As our model molecule, we have used a YFP-fusion of H-Ras, a signaling protein that is anchored in the cytoplasmic leaflet of the plasma membrane. Based on the observed mobility patterns in epidermal cells of the embryos, two fractions of H-Ras molecules were distinguished: a fast- and a slow-diffusing one, which both show confined diffusion. The fast fraction contained the majority of molecules and showed an initial diffusion coefficient that is approximately ten to 15 times higher than that of the slow-diffusing fraction. This fraction showed similar mobility for wild-type H-Ras, the constitutively inactive H-Ras mutant H-Ras^N17^ and the C10H-Ras membrane anchor fused to YFP. However, the fast fraction of a constitutively active H-Ras mutant (H-Ras^V12^) exhibited a higher diffusion coefficient and a larger confinement area. Treatment of the zebrafish embryos with LatB and MBCD significantly increased initial diffusion coefficients of the fast-diffusing fractions for both wild-type H-Ras and its constitutively active, oncogenic mutant, H-Ras^V12^, together with their fraction sizes. Interestingly, the largest variation in these experiments was not found between individual embryos, but between different experimental days and the areas within a single embryo.

In this study, the observation of a fast- and a slow-diffusing fraction of H-Ras molecules that were both confined was highly consistent between experimental models used, i.e. between cultured human embryonic kidney (HEK293T) cells and epidermal cells of zebrafish embryos, between different stages of the zebrafish embryonic development, and between the full-length H-Ras protein, its constitutively active and inactive mutants, and its membrane anchor (C10H-Ras). These fast- and slow-diffusing fractions have been found before in several SMM studies on H-Ras or C10H-Ras (the latter also in zebrafish embryos), with diffusion coefficients comparable to those found in the present study (Lommerse et al., 2004, 2005, 2006; Schaaf et al., 2009). However, confinement of these fractions was not always observed, which in most cases may have been due to the shorter time ranges used in these studies, combined with larger variation or smaller sample sizes.

Although the observed mobility patterns were remarkably stable in our experiments, we did observe some important differences. First, the full-length H-Ras protein displayed lower diffusion coefficients in the epidermal cells of the embryos compared to the diffusion coefficients found in the HEK293T cells. This difference was observed for both fractions of this protein, but was not found for any of the fractions of the C10H-Ras protein. Apparently, the cell type plays a role in determining the mobility of full-length H-Ras, but is less important for the dynamics of the membrane anchor, which suggests that interactions between H-Ras and cytoplasmic components are involved.

Second, the constitutively active mutant protein H-Ras^V12^ showed increased mobility of its fast-diffusing fraction compared to that of the wild-type H-Ras protein, the inactive mutant H-Ras^N17^ and the anchoring domain C10H-Ras, which was reflected in an increased initial diffusion coefficient and a larger confinement area for the active mutant. In a previous study on the mobility of this mutant in cultured mouse embryonic fibroblasts (3T3 cells), this increased mobility was not observed (Lommerse et al., 2006). In addition to the shorter time range, larger variation and smaller sample sizes mentioned earlier as an explanation for such discrepancies, the cell type used may also be a possible factor underlying the absence of this effect in the earlier study. Because wild-type H-Ras, the constitutively inactive mutant H-Ras^N17^ and the anchoring domain C10H-Ras display a similar mobility pattern that is distinct from the pattern observed for the constitutively active mutant H-Ras^V12^, we suggest that the vast majority of H-Ras proteins are in an inactive state in epidermal cells of zebrafish embryos at this stage of development, and that endogenous H-Ras signaling, activated by growth factors such as EGF, is apparently not significantly affecting the dynamics of the H-Ras protein.

This different mobility of the H-Ras^V12^ compared to the inactive forms of H-Ras is in line with the ability of the active mutant to induce oncogenic transformation in a variety of cell types in zebrafish upon overexpression, although phenotypic effects of H-Ras^V12^ were not observed in our study because the formation of tumors does not start before 4 weeks of age (Feng et al., 2010; Michailidou et al., 2009; Santoriello et al., 2010). The alteration in the mobility pattern most likely results from an altered affinity of H-Ras proteins for specific plasma membrane microdomains upon activation, which has already been studied in great detail (Hancock and Parton, 2005; Plowman et al., 2005; Prior et al., 2001, 2003; Zhou et al., 2018). Studies using electron microscopy and other techniques, such as fluorescence recovery after photobleaching, Förster resonance energy transfer and fluorescence correlation spectroscopy, have revealed that the inactive, GDP-loaded H-Ras (as well as its minimal membrane anchor) can form nanoclusters that are located in small (<15 nm) lipid rafts (Plowman et al., 2005; Prior et al., 2001, 2003). These rafts are domains that are smaller than 10-100 nm in size and are dependent on the presence of cholesterol (Garcia-Parajo et al., 2014; Lingwood and Simons, 2010; Nickels et al., 2015; Zhou and Hancock, 2015). Interestingly, a similar dependence on cholesterol was observed for nanoclusters of activated (GTP-loaded) N-Ras, but not for K-Ras nanoclusters or active forms of H-Ras, indicating the formation of alternative nanoclusters in distinct membrane domains that are slightly smaller and cholesterol-independent for these Ras proteins (Plowman et al., 2005; Prior et al., 2001, 2003). Such a preferential dependence on lipids suggests that each type of Ras nanocluster has a unique lipid composition and might, therefore, reflect varying lipid-binding properties between different Ras proteins and between their active and inactive forms (Zhou et al., 2018). It has been demonstrated that the cholesterol-dependent nanoclusters of GDP-bound H-Ras are rich with anionic lipids, such as phosphatidylinositol 4,5-biphosphate (PIP_2_), which enables H-Ras to activate PI3K that binds to PIP_2_ via the P110 subunit (Ghosh et al., 1996; van Rheenen et al., 2005). In addition, phosphatidylserine (PtdSer) and phosphatidylinositol 4-phosphate (PI_4_P) are found in clusters of both GDP- and GTP-bound H-Ras, but only K-Ras localization and clustering on the plasma membrane are sensitive to varying PtdSer concentrations, enabling PtdSer content-sensitive sorting of H-Ras and K-Ras into spatially distinctive lipid assemblies (Zhou et al., 2014). These results show that Ras nanoclusters assemble a distinctive set of plasma membrane phospholipids, which corresponds to their specific effector activation profiles (Zhou and Hancock, 2015; Zhou et al., 2018).

The increased diffusion coefficient and confinement zone of the fast-diffusing fraction observed for H-Ras^V12^ in our study most likely reflect the different diffusion properties of the domains in which the activated protein is preferentially localized. Apparently, the lower affinity for lipid rafts results in an increase in diffusion rate and confinement zone size. These confinement zones in the plasma membrane have been suggested to reflect membrane compartmentalization resulting from the structure of the membrane cytoskeleton, a filamentous actin meshwork associated with the cytoplasmic surface of the plasma membrane, and with various transmembrane proteins anchored to and aligned along the actin filaments (Fujiwara et al., 2002; Kusumi et al., 2010). It could be argued that the diffusion of non-active H-Ras clusters in lipid rafts is even more hindered by these cytoskeleton-based boundaries than by the clusters of activated H-Ras molecules, owing to their larger size. In line with this proposition, the disruption of the cytoskeleton by LatB treatment increased the fast-diffusing fraction's confinement zone size for both the wild-type H-Ras and its constitutively active mutant, H-Ras^V12^.

Furthermore, we demonstrate that MBCD administration also increases the initial diffusion coefficients and size of confinement areas for wild-type H-Ras and H-Ras^V12^. In the presence of this compound, the differences between the mobility patterns of the non-activated and activated form of H-Ras were no longer observed. MBCD depletes cells of cholesterol, thereby disrupting the formation of lipid rafts, which is in line with the largest effect of this compound on the non-active H-Ras form with its larger affinity for lipid rafts. It has previously been shown that LatB disrupts clustering of the non-active H-Ras forms, and it has therefore been suggested that an intact cytoskeleton is required for lipid raft formation (Plowman et al., 2005). Because it has also been shown that cholesterol depletion disrupts the actin cytoskeleton, the interaction between lipid rafts and the cytoskeleton may be bidirectional (Kwik et al., 2003). This interaction is in line with our findings that LatB and MBCD have a similar effect on H-Ras mobility.

In addition, it has been shown that GTP loading increases the probability of H-Ras clusters to be transiently (<1 s) immobilized (Murakoshi et al., 2004), and that interactions with cytoplasmic proteins such as Galectin-1 and Sur-8 are involved in the formation of these immobile H-Ras clusters, which are cholesterol independent and considered to be the sites at which active signaling occurs (Belanis et al., 2008; Hancock and Parton, 2005; Herrero et al., 2016; Li et al., 2000; Shalom-Feuerstein et al., 2008; Zhou et al., 2018). The slow-diffusing fraction observed in our study has a diffusion rate similar to that of a previously identified immobile population (Murakoshi et al., 2004), hence we suggest that the slow-diffusing population contains the actively signaling H-Ras molecules. In contrast to the previous experiments performed in cultured cells, the size of this fraction in the current study was not increased for the active form of H-Ras (Lommerse et al., 2005; Murakoshi et al., 2004). Furthermore, the size of the slow-diffusing H-Ras fraction decreased significantly for both wild-type H-Ras and H-Ras^V12^ upon treatment with LatB and MBCD, which underlines the indispensable role of the structure and composition of the plasma membrane in the process of H-Ras cluster immobilization.

Using the mobility data obtained in zebrafish embryos, we studied the factors that contributed to the variation in these data. Interestingly, differences between individual embryos had only a modest effect on the overall variation in the results. However, different experimental days and different areas of the embryonic epidermis that we imaged both appeared to be factors that showed a large contribution to the overall variation in the data. The area that we imaged had a size of 16.6×16.6 µm, which is approximately the area of a single cell. Thus, we assume that cell-to-cell variation within the epidermal tissue was a main source of the observed variation. This also indicates that the cellular context largely determines H-Ras mobility, and that differences between cells within the epidermal tissue of one individual embryo are larger than the differences in average cellular context in the tissue between embryos. The observation that the membrane anchor C10H-Ras fused to YFP shows a mobility pattern similar to that of the full-length YFP-H-Ras fusion protein indicates that the structure of the inactive, GDP-loaded protein does not define the mobility patterns and that, therefore, interactions with structural elements of the cell, such as specific membrane domains and the cytoskeleton, are determining the dynamics of H-Ras in the absence of an activating stimulus. A role of the endogenous zebrafish H-Ras signaling pathway in this cell-to-cell variability cannot be excluded.

In addition to the cell-to-cell variation, day-to-day variation also contributed to the total variability. As there was not much variability originating from the use of different zebrafish embryos, we suggest that these differences may stem from minimal changes in mounting and imaging procedures, even though the experiments were performed by the same researcher. This source of variation may, in future studies, be minimized by imaging an identical, well-defined region of the larval tail fin. Furthermore, small differences in treatment conditions might affect embryonic development, alter complex metabolic pathways and induce stress responses, which may cause the observed differences in the values of the measured dynamic parameters on different experimental days. Finally, the AB/TL zebrafish wild-type strain that was used is not an inbred strain, and its genetic heterogeneity might underlie batch-to-batch variation. This source of variation can, in future studies, be minimized by utilizing embryos from an isolated and identical couple of adult zebrafish.

In conclusion, in the present study, we have used a previously developed TIRFM-based approach to perform SMM in an intact vertebrate organism. Our study confirms that this technology is highly useful for studying the molecular behavior of individual receptors and signaling molecules, and enables further *in vivo* studies to unravel exact molecular mechanisms governing molecular interactions and their role in physiological and pathological processes, such as skin cancer, wound healing or tissue regeneration (Kaufman et al., 2016; Kawakami et al., 2004). We have studied the mobility pattern of H-Ras in the epidermis of living zebrafish embryos, and our data show the consistent presence of a fast- and a slow-diffusing fraction of H-Ras molecules, which both show confinement. Activation of H-Ras changes the mobility and confinement of the fast-diffusing fraction, which is dependent the presence of specific membrane microdomains. Interestingly, our data demonstrate that epidermal tissue shows a large degree of heterogeneity between individual cells.

## MATERIALS AND METHODS

### Zebrafish

Wild-type ABTL zebrafish (*Danio rerio*) and zebrafish from the transgenic line *Tg(bactin:GFP-C10H-Ras)^vu119^* were grown and maintained according to standard protocols (http://ZFIN.org), exposed to a 14 h light and 10 h dark diurnal cycle at 28°C. Fertilization was performed by natural spawning at the beginning of the light period. Eggs were collected and raised in egg water (60 µg/ml Instant Ocean Sea Salts, Cincinnati, OH, USA) at 28°C. All experiments performed on living zebrafish embryos were done in compliance with the directives of the local animal welfare committee of Leiden University.

### Cell cultures, transfection and fixation

In all cell culture experiments, authenticated human embryonic kidney cells (HEK293T) were used (ATCC, Manassas, VA, USA). Cells were screened for mycoplasma contamination on a monthly basis. Cells were cultured in Dulbecco's modified Eagle medium (DMEM; Invitrogen, Waltham, MA, USA) supplemented with penicillin and streptomycin (10 µg ml^−1^, Invitrogen), Glutamax (10 µg ml^−1^, Invitrogen) and 10% fetal calf serum (Invitrogen) at 37°C in a humidified atmosphere containing 5% CO_2_. Cells were passaged every 3-4 days and kept in use for a maximum of 12 passages. Before transfection, cells were transferred onto a sterile, glass coverslip (diameter 25 mm; Marienfeld, Lauda-Königshofen, Germany) placed in a well of a six-well plate. At a confluency level of 20-30%, cells were transfected with 1 µg DNA per well, using FuGENE 6, according to the manufacturer's protocol (Roche Molecular Biochemicals, Indianapolis, IN, USA). The transfection efficiency, determined by fluorescence microscopy screening at 48 h after transfection (EVOS M7000 Cell Imaging Systems, Thermo Fisher Scientific, Waltham, MA, USA), was ∼30%. Cells were imaged at least 4 days post-transfection to lower the expression level of the YFP-fused proteins and to be able to efficiently observe single molecules. Prior to the SMM experiments, DMEM was replaced by phosphate-buffered saline (PBS; 150 mM NaCl, 10 mM Na_2_HPO_4_/NaH_2_PO_4_, pH 7.4) kept at room temperature. Finally, the coverslips with cells were mounted on a microscope holder, and 500 µl room-temperature PBS was pipetted onto the cells. For single-step photobleaching experiments, transfected cells were fixed using 4% paraformaldehyde in PBS overnight, at 4°C. Immediately before the experiments, the cells were washed three times, 5 min each time, with PBS. Finally, the cells were rinsed twice with PBST (PBS with Tween 20, 0.1%), for 5 min each time.

### Microinjection of DNA in zebrafish embryos

The cDNAs encoding human YFP-C10H-Ras, YFP-H-Ras, YFP-H-Ras^V12^ and YFP-H-Ras^N17^ were cloned from pcDNA3.1(+) mammalian expression plasmids into pCS2(+) plasmids, which then served as vectors for expression of the fluorescent proteins of interest. DNA plasmid microinjections were performed at a concentration dose of 30 pg per embryo at the one- to two-cell stage, resulting in mosaic expression of the fluorescent protein in the zebrafish embryos at later stages. Microinjections were done using a Femtojet microinjector (Eppendorf, Hamburg, Germany) and a micromanipulator with pulled microcapillary pipettes. The procedure of microinjections was controlled under a stereomicroscope (M165C, Leica Microsystems, Wetzlar, Germany). Injected eggs were then left to develop in an incubator at 28°C. Viability and development of the eggs after microinjections was checked on a daily basis using fluorescence stereo- or confocal microscopy*.*

### Treatment of zebrafish embryos with LatB and MBCD

Inhibition of actin polymerization was induced using a protocol described previously (Kugler et al., 2019). LatB (Sigma-Aldrich, St Louis, MA, USA) was dissolved in 96% ethanol to a 500 µM stock concentration. At 48 hpf, zebrafish embryos were dechorionated and treated with 100 nM LatB, dissolved in egg water, for 1 h. A control group was treated with diluted vehicle (0.02% ethanol) only. After LatB treatment, zebrafish were immediately transferred and imaged under the SMM setup. Treatment with MBCD was based on protocols described before (Bello-Perez et al., 2020; da Silva et al., 2019). MBCD (Sigma-Aldrich) was dissolved in PBS (pH 7.4) to a stock concentration of 400 nM. Analogously to the LatB treatment, dechorionated zebrafish embryos were treated with MBCD, at a final concentration of 40 nM, at 48 hpf. A control group was treated with diluted vehicle (10× diluted PBS in egg water) only. After MBCD treatment, zebrafish were immediately transferred and imaged under the SMM setup.

### Fluorescence stereomicroscopy

For screening zebrafish embryos expressing YFP-fused proteins, a Leica M205FA fluorescence stereomicroscope (Leica Microsystems) was used. Images of the zebrafish embryos were taken using a Leica DFC 345FX camera.

### Confocal laser-scanning microscopy

A Leica SPE confocal laser-scanning microscope (Leica Microsystems) was used to investigate the fluorescent signal in the zebrafish embryo. Excitation was done using an argon laser at 514 nm. Images were obtained using 20× and 40× non-immersion objectives (0.70 NA and 0.80 NA, respectively) and a 63× water immersion objective (1.20 NA).

### TIRFM

Glass coverslips were washed with 99% ethanol (twice), HPLC-grade water (twice), KOH (1 M, twice) and acetone (99%, three times). Each wash was followed by a 30-min-long sonication period at 50°C. If not used immediately, the coverslips were stored in acetone. Prior to the mounting of the zebrafish embryos, the glass coverslips were coated with 50 µg ml^−1^ poly-L-lysine (Sigma-Aldrich) for 5 min, followed by a double wash with deionized water and drying with nitrogen gas. Two-day-old zebrafish embryos were equilibrated at room temperature for 1 h, anesthetized with 0.02% aminobenzoic acid ethyl ester (tricaine, Sigma-Aldrich) and dechorionated using tweezers. Subsequently, a single zebrafish embryo was placed on a coverslip with a lateral side against the coverslip, while excess water was aspirated. The tail of the embryo was pressed against the surface of the coverslip by a thin agarose sheet (2%, thickness 0.75 mm). A drop of egg water was added to cover the rest of the embryo's body. All the TIRFM measurements were performed at room temperature. The coverslip with the embryo was placed on a microscope holder and mounted on the TIRFM setup. This setup was a custom-made microscope with a 100× oil-immersion objective (1.45 NA; Nikon, Tokyo, Japan). Excitation was performed using a 515 nm laser (iChrome MLE, Toptica Photonics, Germany); the field of view was set to a 100×100 pixels region with a pixel size of 166 nm, and the laser power equaled to 20% of the maximal laser power (40 mW). The incident laser beam was set at the critical angle against the coverslip–water interface, thus being totally reflected and creating the evanescent wave for excitation of fluorophores close to the coverslip–sample interface. Emission light was filtered using a long pass filter (ET5701p, Chroma Technology, Bellows Falls, VT, USA), and image sequences were collected using an on-chip multiplication gain CCD camera (model 512B, Cascade, Roper Scientific, Tucson, AZ, USA). Each image sequence contained 1200 frames separated by a 25 ms time lag, resulting in the total acquisition time of 30 s.

### Analysis of protein diffusion patterns

The analysis of the position of individual molecules was done as described previously. Fluorescence intensity signals corresponding to YFP molecules were fitted to a two-dimensional Gaussian surface, using previously custom-developed software (Groeneweg et al., 2014; Lommerse et al., 2004, 2005). The software, in the form of a code written in MATLAB programming environment (MathWorks, Natick, MA, USA), can be obtained from T.S. Subsequently, the corresponding signals were filtered based on their FWHM value and their intensity count, based on the microscopy setup. The FWHM and intensity threshold values were obtained in the single-step photobleaching experiments (performed using fixed HEK293T cells), by averaging the Gaussian distributions of 20 different YFP molecules in the last step prior to the photobleaching-induced final intensity drop (using TrackMate plug-in, ImageJ). The location of a molecule was defined by the center of the Gaussian curve. Positional accuracy *dx* of the peak localization equaled ∼22 nm (Groeneweg et al., 2014; Schütz et al., 1997).

To study the mobility pattern of the proteins, PICS software was used, which has been previously described (Semrau and Schmidt, 2007). The PICS software, in the form of a code written in MATLAB programming environment, can be obtained from T.S. or Stefan Semrau (Leiden Institute of Physics, Leiden University) (Semrau and Schmidt, 2007). A multistep analysis was performed for each image sequence acquired, yielding information for five different time lags of 25, 50, 75, 100 and 125 ms. In PICS analysis, individual particles are not tracked, but correlations between the location of molecules in consecutive frames are determined. This way, cumulative probability distributions of the squared displacements were generated for each of the time lags, and fitted to a one- or two-population model. The former is described by the equation:
(1)

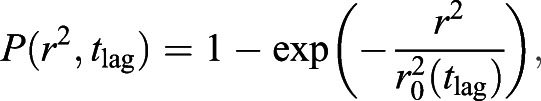
which describes the probability that a particle exhibiting Brownian motion at the arbitrary origin is found within a circle of a radius *r* at the time lag *t*_lag_, and its mean squared displacement equals 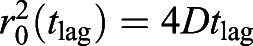. However, in case that the populations of the molecules can be differentiated into two different populations, Eqn 1 is transformed into:
(2)

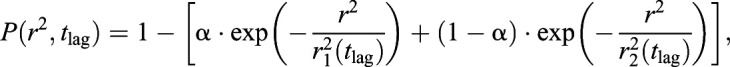
where the mean squared displacement of fast-diffusing and slow-diffusing populations are denoted by 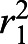 and 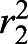, and their relative sizes by α and 1−α, respectively (Schaaf et al., 2009). Hence, α represents the fraction size of the fast-diffusing H-Ras molecules and is represented as a percentage of the total population.

To examine whether any of these populations confine to a certain area, the values of 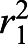 and 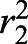 were plotted against the time lag. For the larger time lags the variance increases due to the smaller number of statistically independent measurements of *r*^2^ (Kusumi et al., 1993). The positional accuracy *dx* led to a constant offset in *r*^2^ of 4·(*dx*)^2^, which, in our case, equaled 0.0049 µm^2^. The plots were fitted by a free Brownian diffusion model, with a diffusion coefficient *D* in the fitted equation 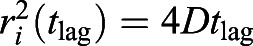, or by a confined diffusion model described by the equation:
(3)

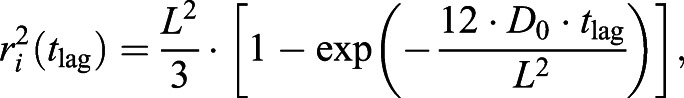
in which the molecules move freely with an initial diffusion coefficient *D*_0_, but are confined by impermeable barriers within an area described by a square of a side length *L*.

### Experimental design

In every experiment, three different zebrafish embryos were selected for the TIRFM imaging. In each of the selected embryos, at least three separate areas were imaged. The data shown reflect a minimum of three independent experiments, each performed on three different days. Six independent experiments were done for YFP-C10H-Ras (18 individual embryos), and three for the wild-type YFP-H-Ras, YFP-H-Ras^N17^ and YFP-H-Ras^V12^ (nine individual embryos per H-Ras construct). For studying the influence of the developmental stage on the YFP-C10H-Ras dynamics, an analogous design was implemented (three experiments, three individual embryos per each experiment), yet the embryos were selected and imaged at 48, 56, 72 and 80 hpf. For studying the influence of zebrafish treatment with LatB and MBCD on the YFP-C10H-Ras and YFP-H-Ras^V12^ dynamics, an analogous design was implemented (three experimental days, three individual embryos selected per vehicle, LatB treatment and MBCD treatment groups). In the case of transfected HEK293T cells, three independent experiments were performed for both YFP-C10H-Ras and wild-type YFP-H-Ras. In each of those experiments, three different coverslips with growing cells were selected from a six-well plate, and at least three different cells were imaged on each of the coverslips.

### Statistical analysis

Values of the fast-diffusing population sizes and squared displacements were averaged per experimental day for each time lag in all individual groups (i.e. YFP-C10H-Ras, YFP-H-Ras, YFP-H-Ras^N17^ and YFP-H-Ras^V12^). Statistical analysis of the data (presented in Table 2) was performed for the experimental time lag of 25 ms by comparing: (1) results obtained for HEK293T cells expressing YFP-C10H-Ras and wild-type YFP-H-Ras; (2) results obtained from zebrafish embryos expressing YFP-C10H-Ras at 48, 56, 72 and 80 hpf; (3) results obtained from zebrafish embryos expressing wild-type YFP-H-Ras, YFP-H-Ras^N17^ and YFP-H-Ras^V12^; and (4) results obtained from zebrafish embryos expressing wild-type YFP-H-Ras and YFP-H-Ras^V12^ after treatment with LatB and MBCD. In addition, a comparison between biological models was performed, using results obtained for YFP-C10H-Ras and wild-type YFP-H-Ras for both zebrafish embryos and HEK293T cells. Intragroup variability analysis was carried out between all experimental groups belonging to the same biological model and construct expressed. Initial diffusion coefficients and confinement area sizes were obtained through a confined model fit, by pooling and averaging values of mean squared displacements for each individual H-Ras construct, biological model, treatment and time post-fertilization.

In order to check whether data were normally distributed, a Shapiro–Wilk statistical test was performed. Significance of the results was performed using an unpaired Student's *t*-test for comparison of means between two, normally distributed, groups. When multiple groups were compared, a one-way ANOVA was implemented with a Tukey range test for post-hoc analysis. Ultimately, the source of potential variability of the data was examined using a hierarchical linear model to account for nested structure of the data (i.e. different areas within an embryo, different embryos within an experimental day). The values of the *r*^2^ were logarithm transformed, and those of the α were logit transformed, to meet the hypotheses of the statistical model.

This article is part of a collection ‘The RAS Pathway: Diseases, Therapeutics and Beyond’, which was launched in a dedicated Special Issue guest edited by Donita Brady and Arvin Dar. See related articles in this collection at https://journals.biologists.com/dmm/collection/5089/The-RAS-Pathway.

## Supplementary Material

Supplementary information
