# Supplementary material for: Analysis of the H-Ras mobility pattern in vivo shows cellular heterogeneity inside epidermal tissue
Source: Dis Model Mech. 2022 Feb 24;15(2):dmm049099. doi: 10.1242/dmm.049099 (PMC8891639; doi:10.1242/dmm.049099)
Supplement: Supplementary information [file dmm-15-049099-s1.pdf]

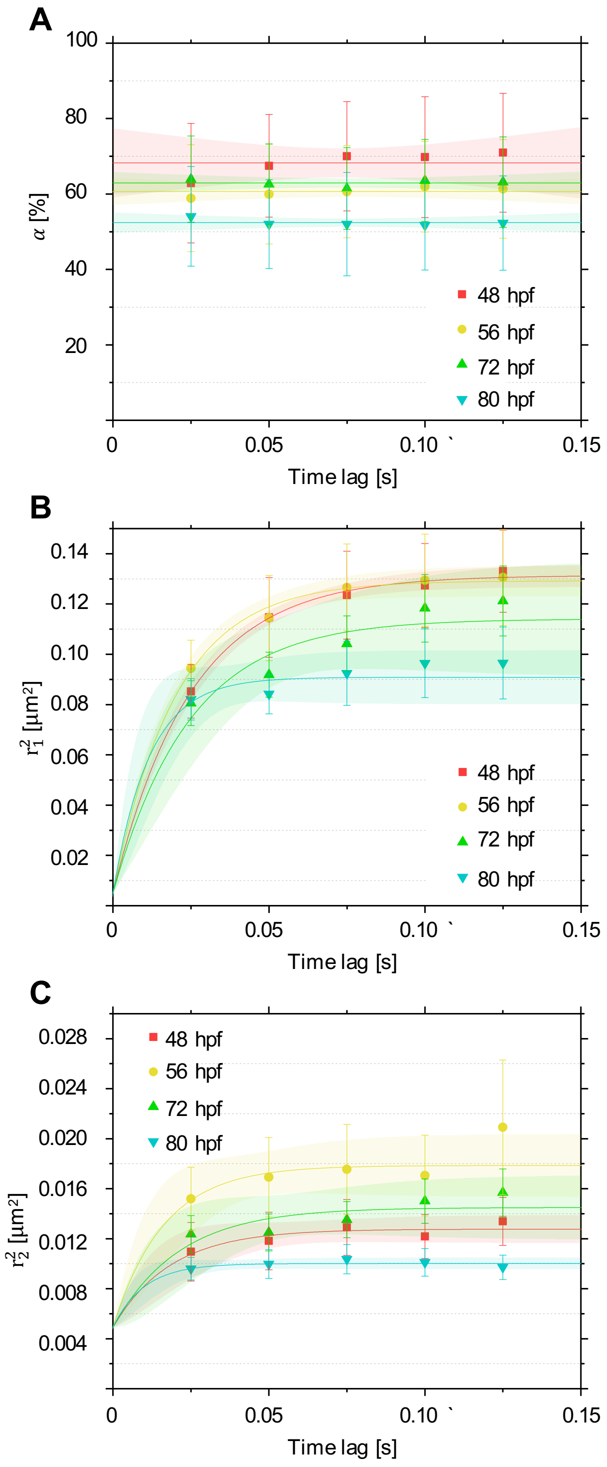

A
100
80
60
40
20
α [%]
48 hpf
56 hpf
72 hpf
80 hpf
0
0.05
0.10
0.15
Time lag [s]
B
0.14
0.12
0.10
0.08
0.06
0.04
0.02
r_1^2 [μm²]
48 hpf
56 hpf
72 hpf
80 hpf
0
0.05
0.10
0.15
Time lag [s]
C
0.028
0.024
0.020
0.016
0.012
0.008
0.004
r_2^2 [μm²]
48 hpf
56 hpf
72 hpf
80 hpf
0
0.05
0.10
0.15
Time lag [s]

Fig. S1. Mobility patterns of YFP-C10H-Ras in epidermal cells of the zebrafish embryos at different developmental stages. (A) Fraction size of the fast-diffusing population (α), plotted against the time lag. **(B)** Mean squared displacements plotted against the time lag for the fast-diffusing fraction (${r_1}^2$). **(C)** Mean squared displacements plotted against the time lag for the slow-diffusing fraction (${r_2}^2$). Results of the fits are summarized in Table 1. To establish the values of dynamic parameters, 3 different embryos were imaged on each of the 3 different experimental days at 48-, 56-, 72-, and 80-hour post fertilization. Each datapoint is presented in the form of a mean $\pm$ s.e.m., and the 95% c.i. of the mathematical fit is shown. Shapiro-Wilk statistical test was performed to check for normality of the data set. Statistical analysis was performed using a one-way ANOVA (P-value P(α, ${r_1}^2$, ${r_2}^2$) > 0.05 at a t_{lag} of 25 ms) with a Tukey's range post-hoc test.
